# Genomic epidemiological analysis of SARS-CoV-2 household transmission

**DOI:** 10.1099/acmi.0.000252

**Published:** 2021-07-19

**Authors:** Daniel Hare, Gabriel Gonzalez, Jonathan Dean, Kathleen McDonnell, Michael J. Carr, Cillian F. De Gascun

**Affiliations:** ^1^​ National Virus Reference Laboratory (NVRL), School of Medicine, University College Dublin 4, Belfield, D04 V1W8, Dublin, Ireland; ^2^​ International Collaboration Unit, Research Center for Zoonosis Control, Hokkaido University, N20 W10 Kita-ku, Sapporo, 001-0020, Japan; ^3^​ Public Health Department, Health Service Executive South East, Kilkenny, Ireland

**Keywords:** COVID-19, family cluster, outbreak investigation, SARS-CoV-2, Whole-Genome-Sequencing

## Abstract

Family clusters have contributed significantly to the onward spread of SARS-CoV-2. However, the dynamics of viral transmission in this setting remain incompletely understood. We describe the clinical and viral-phylogenetic characteristics of a family cluster of SARS-CoV-2 infections with a high attack rate, and explore how whole-genome sequencing (WGS) can inform outbreak investigations in this context. In this cluster, the first symptomatic case was a 22-month-old infant who developed rhinorrhoea and sneezing 2 days prior to attending a family gathering. Subsequently, seven family members in attendance at this event were diagnosed with SARS-CoV-2 infections, including the infant described. WGS revealed indistinguishable SARS-CoV-2 genomes recovered from the adults at the gathering, which were closely related genetically to B.1 lineage viruses circulating in the local community. However, a divergent viral sub-lineage was recovered from the infant and another child, each harbouring a distinguishing spike substitution (N30S). This suggested that the infant was unlikely to be the primary case, despite displaying symptoms first, and additional analysis of her nasopharyngeal swab revealed a picornavirus co-infection to account for her early symptoms. Our findings demonstrate how WGS can elucidate the transmission dynamics of SARS-CoV-2 infections within household clusters and provide useful information to support outbreak investigations. Additionally, our description of SARS-CoV-2 viral lineages and notable variants circulating in Ireland to date provides an important genomic-epidemiological baseline in the context of vaccine introduction.

## Introduction

Family clusters of infections have represented the majority of SARS-CoV-2 outbreaks in Ireland to date [1]. Whole-genome sequencing (WGS) has been crucial in elucidating the dynamics of virus transmission in many settings, as well as monitoring the evolution of SARS-CoV-2 [2], although few studies have examined family clusters in detail using a genomics approach. We describe the clinical, laboratory and viral phylogenetic characteristics of a family cluster of SARS-CoV-2 infections in the context of national SARS-CoV-2 genomic data, in which WGS clarified the chain of transmission and refuted the standard epidemiologically-generated hypothesis.

## Methods

### Case definition and molecular diagnostics

Cases were confirmed by detection of SARS-CoV-2 RNA by real-time reverse transcriptase PCR (qRT-PCR) performed on oro- and nasopharyngeal swabs. In the context of viral genomic epidemiological surveillance, specimens in which SARS-CoV-2 RNA was detected were sent to the National Virus Reference Laboratory (NVRL), University College Dublin (UCD) for WGS. Testing for an additional 21 viral and bacterial respiratory pathogens was performed using the NxTAG *Respiratory Viral* Panel FAST v2 (Luminex). This included Rhinovirus/Enterovirus, Influenza A and B, Parainfluenza 1–4, Human metapneumovirus, Bocavirus, Adenovirus, RSV A and B, seasonal coronaviruses OC43, HKU1, NL63 and 229E, *Chlamydophila pneumoniae* and *Mycoplasma pneumoniae*.

### Whole-genome sequencing and bioinformatics

SARS-CoV-2 genome sequences were generated from laboratory-confirmed cases referred to the NVRL, Dublin, between 25 June and 15 November 2020, with Ct values less than 30. This included the family cluster described. Viral RNA was extracted and purified using the Roche MagNa Pure 96 instrument. A tiled amplicon approach following the ARTIC v3 sequencing protocol [3] was employed, using the MinION platform (Oxford Nanopore Technologies, ONT), and assembled with the artic-ncov2019 pipeline [4]. Sequences were multiple sequence aligned against the reference genome sequence (GenBank accession number: MN908947) with MAFFT [5], and lineage identification was according to the PANGOLIN nomenclature developed by Rambaut *et al.* [6]. Maximum-likelihood phylogenetic trees were built with RAxML [7]. All virus genome sequences were deposited and are publicly available in the Global Initiative on Sharing All Influenza Data (GISAID) database, with accession IDs outlined in Table S1 (available in the online version of this article). Sequences corresponding to the family cluster include accession IDs: EPI_ISL_578327, EPI_ISL_578240, EPI_ISL_578325, EPI_ISL_578235, EPI_ISL_578324, EPI_ISL_578326 and EPI_ISL_578321.

## Results

### Outbreak description

#### Cases 1 and 2

On 13 August 2020, a 22-month-old girl (case 1) who lived with her parents in the south-east of Ireland, developed purulent rhinorrhoea and sneezing, followed by diarrhoea shortly thereafter. Two days later, she was brought to a family gathering which lasted two-and-a-half hours, where she had close contact with nine family members. This included her mother, both maternal grandparents, two uncles and their partners, and two cousins aged 1 and 3 years. Upon leaving the gathering, the mother of case 1 (case 2) experienced pronounced fatigue, headache and mild diarrhoea, with a low-grade fever of 37.8 degrees Celsius. Case 2 sought medical attention from her General Practitioner and was referred for testing for suspected SARS-CoV-2 infection on 17 August, with viral RNA detected by qRT-PCR (Cycle threshold, Ct 15.7).

As close contacts of case 2, all who attended the family gathering were referred for SARS-CoV-2 RNA testing, which took place on 20 August. This included case 1, who also had SARS-CoV-2 RNA detected (Ct 25). Case 1 experienced persistent nasal congestion for a total of 14 days, whereas case 2 developed mild shortness of breath and anosmia between 22 August and 24 August: this improved without the need for specific medical treatment.

#### Cases 3 to 7

**Fig. 1.**
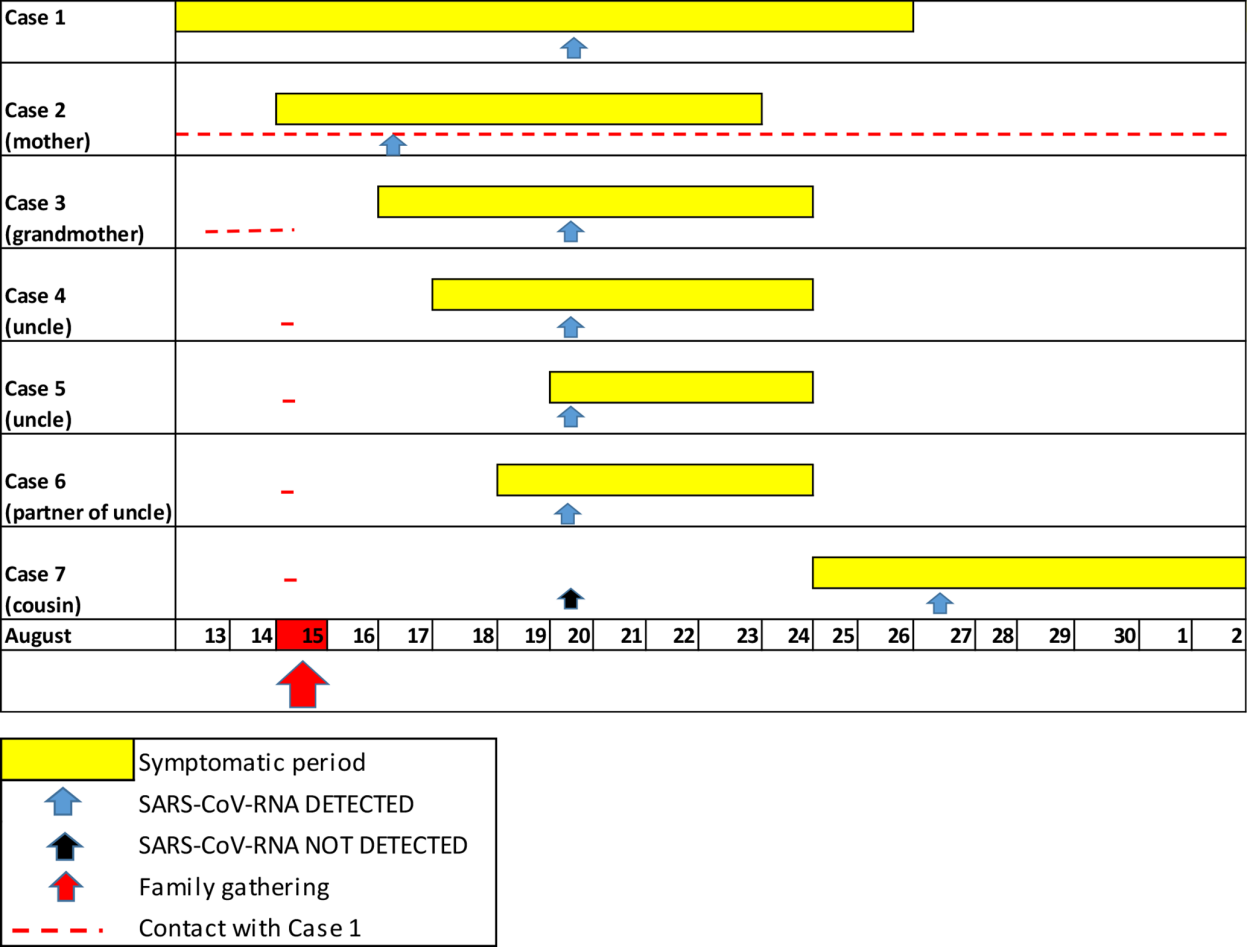
Timeline of symptoms relative to SARS-CoV-2 qRT-PCR tests and contact with case 1. Of the ten family members in attendance at the family gathering, seven had SARS-CoV-2 RNA detected by qRT-PCR: RNA was not detected in three.

**Fig. 2.**
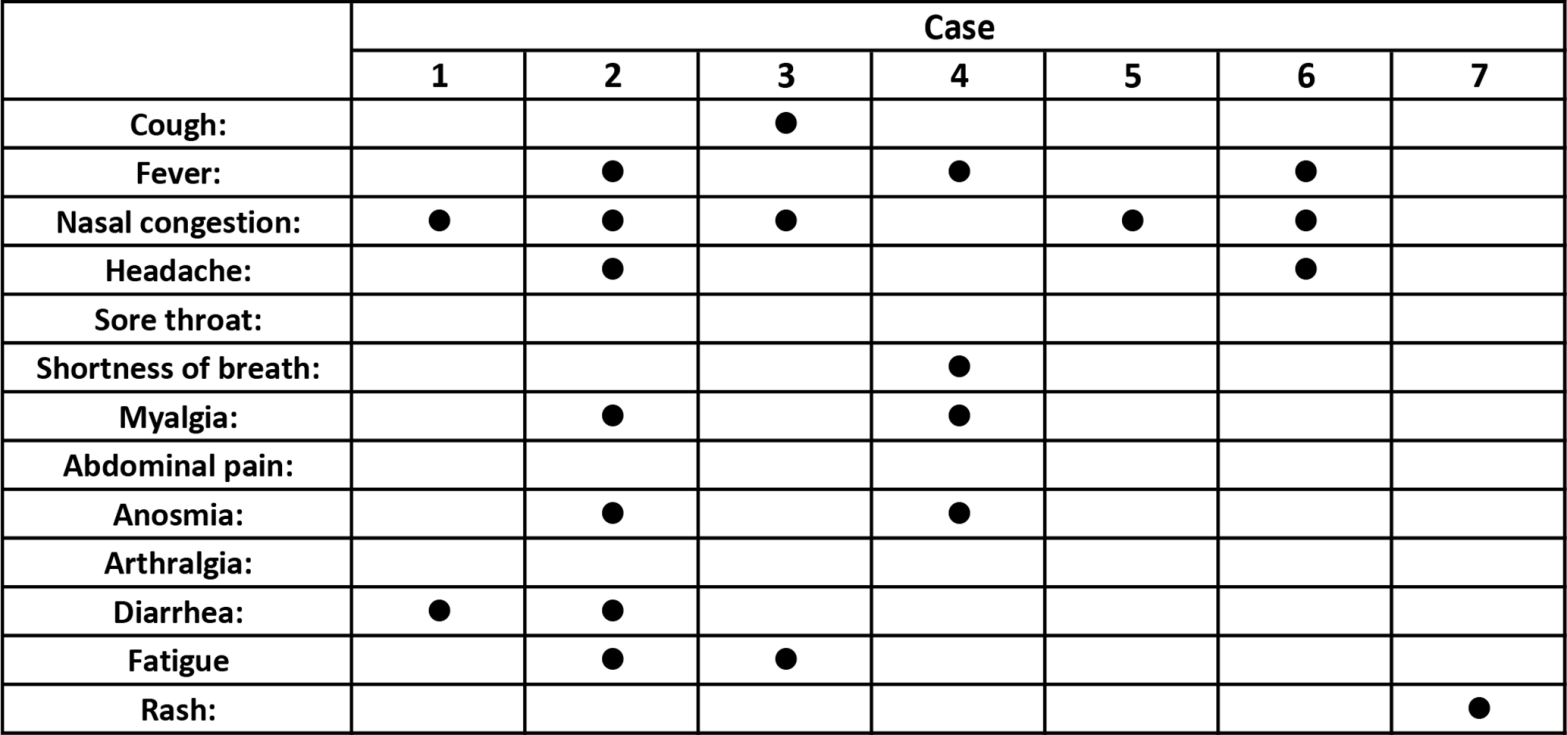
Range of symptoms reported by cases 1–7.

Five of eight other family members who were present at the gathering on 15 August yielded 'SARS-CoV-2 RNA detected' results (Ct range 16–27) when tested on 20 August. All had close contact with case 1 at the event and had developed mild symptoms in the days that followed. The timeline of symptoms for each case is depicted in Fig. 1, with the range of symptoms observed in Fig. 2 and qRT-PCR results with Ct values shown in Table 1.

**Table 1.** Real-time qRT-PCR results, including cycle threshold (Ct) levels for SARS-CoV-2 targets, corresponding to the household outbreak

Case	Sample date	SARS-CoV-2 PCR	E gene	S gene	N gene	NxTAG *respiratory viral Panel*
Case 1	20/08/2020	*RNA DETECTED*∗	n/a	n/a	25	*RHINOVIRUS/ENTEROVIRUS RNA DETECTED*
Case 2	17/08/2020	*RNA DETECTED*†	15.7	15.8	n/a	*VIRAL NUCLEIC ACID NOT DETECTED*
Case 3	20/08/2020	*RNA DETECTED*∗	n/a	n/a	18.8	*VIRAL NUCLEIC ACID NOT DETECTED*
Case 4	20/08/2020	*RNA DETECTED*∗	n/a	n/a	21	*VIRAL NUCLEIC ACID NOT DETECTED*
Case 5	20/08/2020	*RNA DETECTED*∗	n/a	n/a	19	*VIRAL NUCLEIC ACID NOT DETECTED*
Case 6	20/08/2020	*RNA DETECTED*∗	n/a	n/a	16	*VIRAL NUCLEIC ACID NOT DETECTED*
Case 7	20/08/2020	*RNA NOT DETECTED*‡	n/a	n/a	n/a	*VIRAL NUCLEIC ACID NOT DETECTED*
	27/08/2020	*RNA DETECTED*‡	n/a	n/a	27	*VIRAL NUCLEIC ACID NOT DETECTED*

∗IDEXX SARS-CoV-2 (COVID-19) RealPCR Test.

†Altona RealStar SARS-CoV-2 RT-PCR Kit.

‡GSD NovaPrime SARS-CoV-2.

E, Envelope; S, Spike; N, Nucleoprotein.

Case 3, a 65-year-old female with type 2 diabetes mellitus and grandmother of case 1, developed a non-productive cough on 17 August; this was followed by nasal congestion and fatigue for 7 days. She had ten hours of close contact with case 1 between 14 and 16 August, which included the family gathering.

Case 4, a 35-year-old uncle of case 1, developed myalgia, fatigue and subjective fevers between 18 August and 20 August; this was followed by mild shortness of breath and anosmia lasting for 2 days, from 22 August to 24 August. Of note, he was at the family event for a period of only 20 minutes but recalled sharing an ice cream with case 1 during that time.

Case 5, another uncle of case 1, developed mild nasal congestion on 20 August, lasting for 5 days only, while his partner, case 6 (who was also present at the family gathering), developed headache and nasal congestion on 19 August for a period of 5 days.

Case 7, a 3-year-old male cousin of case 1 (and son of case 4), developed a rash on 25 August, ten days after the family gathering. He had not attended a crèche in the 14 days preceding the gathering; however, he shared a household with case 4. He had regular contact with case 3 between 15 August and 17 August but no further contact with case 1 after the event on 15 August. When initially tested on 20 August his qRT-PCR test result was 'SARS-CoV-2 RNA not detected': subsequently however, SARS-CoV-2 RNA was detected on a repeat test 7 days later (Ct 27).

Two adults present at the event were asymptomatic and had two consecutive qRT-PCR tests with SARS-CoV-2 RNA not detected, whereas one of the children present at the gathering had symptoms of nasal congestion, but also tested negative on 20 August. Based on the number of susceptible individuals and assuming a single introduction of SARS-CoV-2 into this family at the event, the secondary attack rate was 67 % (six out of nine susceptible family members infected; Wilson 95 % confidence interval: 35–88 %). None of the cases – symptomatic or otherwise – required hospitalization or medical treatment, with nasal congestion a predominant manifestation in five out of seven cases.

### Outbreak control measures

In accordance with national Public Health guidance, all of the adults in attendance at the gathering had been working from home, and none had travelled outside of Ireland in the preceding months. All restricted their movements for 14 days after diagnosis and close contacts were followed actively by Public Health. Screening of close contacts, which included mass testing of the crèche attended by case 1, revealed no further SARS-CoV-2 infections.

### Whole-genome sequencing

**Fig. 3.**
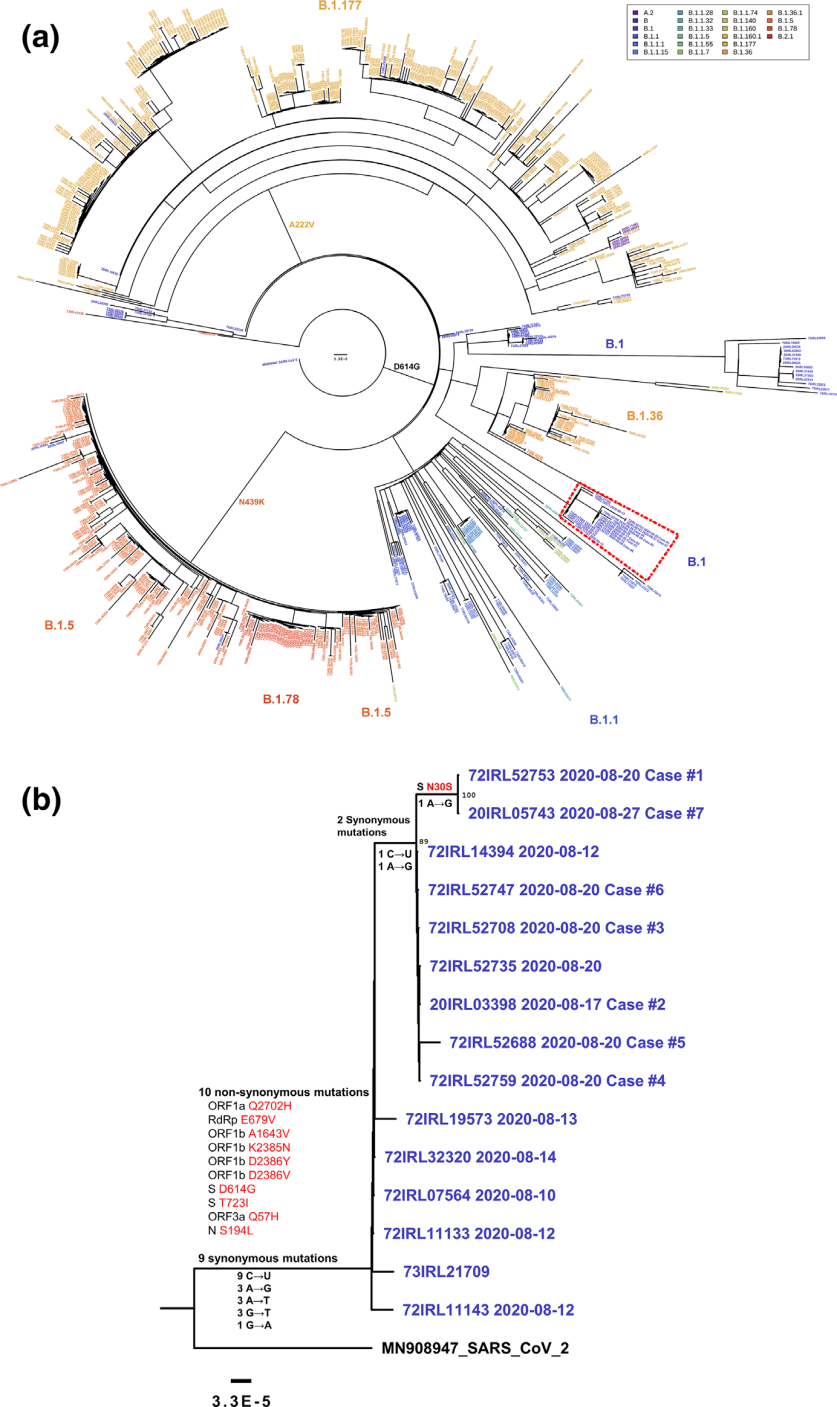
(a) Phylogeny of Irish community SARS-CoV-2 sequences (*n*=674), including the assigned viral lineages and notable spike variants observed. SARS-CoV-2 whole-genome sequences were generated from laboratory-confirmed cases between 25 June and 15 November 2020. This family cluster is highlighted in the red dashed box. (b) Maximum-likelihood phylogenetic tree representing the family cluster, rooted to the reference strain Wuhan-Hu-1 (GenBank accession number: MN908947). Distinctive mutations are shown next to the branches, non-synonymous mutations and the mutated ORFs are detailed. A combination of two synonymous mutations separate the cluster from other B.1 lineage genomes from the region, with the acquisition of a further non-synonymous A-to-G mutation in the ORF encoding the spike protein (S:N30S) identified in Cases 1 and 7.

High-quality consensus genome sequences were generated from SARS-CoV-2 RNA extracted from samples obtained from the family. A maximum-likelihood phylogenetic tree rooted to the Wuhan reference strain (GenBank accession number: MN908947), reconstructing these sequences was created, and is shown in Fig. 3 (b), along with a phylogeny of Irish community SARS-CoV-2 genomes (*n*=674) from laboratory-confirmed cases sequenced at the NVRL between 25 June and 15 November 2020. Viral genomes recovered from all cases in this cluster were assigned to the PANGOLIN SARS-CoV-2 B.1 lineage with high confidence, and were shown to be closely related to other SARS-CoV-2 genomes circulating contemporaneously from the south-east of the country. However, the family sequences diverged by a unique combination of two synonymous mutations from others sampled in that region (C-to-U at nucleotide 10 030 and A-to-G at nucleotide 21 625, relative to the reference). Furthermore, the genomes sequenced from cases 2 to 6 were genetically indistinguishable, indicating a single point source of infection to the family from a viral lineage circulating locally, based on genetic relatedness of this clade to B.1 lineages in community circulation. Interestingly, the virus sequences recovered from the infants at the family gathering (cases 1 and 7) were found to have diverged from the rest of the family by the acquisition of an additional nonsynonymous A-to-G mutation (nucleotide 21 651 in the reference) changing residue 30 of the spike protein (S:N30S) (Fig. 3). Interrogation of the GISAID database revealed an identical A-to-G mutation at the same nucleotide position resulting in S:N30S in just one other sequenced genome submitted to the platform to date (hCoV-19/Panama/335944/2020, GISAID accession: EPI_ISL_496830); however, this sequence belongs to the A.2 lineage implying independent acquisition of the N30S mutation in both lineages.

Despite displaying symptoms earlier than any other family member, these viral genomic findings suggested that the 22-month-old infant (case 1) became infected after the adults and was therefore unlikely to be the primary case. This prompted further analysis of her original specimen, which revealed the presence of picornavirus (Rhinovirus/Enterovirus) RNA at the time of testing, suggesting that a co-infection may have accounted for her early symptoms. However, given that an identical viral genome with the same S:N30S amino acid substitution was also recovered from her cousin, who had SARS-CoV-2 RNA detected 1 week later and no further contact with case 1, supported the hypothesis that case 1 transmitted to case 7 at the family event. All family members who had SARS-CoV-2 RNA detected were subsequently tested for the presence of other viral respiratory pathogens. Other than case 1, none returned positive results (Table 1).

## Discussion

### SARS-CoV-2 transmission within families

Since its recognition as a novel pathogen in late 2019, SARS-CoV-2 has been shown to transmit readily within households [8], and SARS-CoV-2 clusters within private homes have represented the majority of community outbreaks recorded in Ireland to date [1]. Although meta-analyses have estimated secondary attack rates within families to be 18.1–18.8 % [9, 10], a recent prospective study found transmission rates to be as high as 53 % [11]; considerably higher than outside of this environment.

The relative contribution of young children in the onward transmission of SARS-CoV-2 in the context of household clusters is debated. In the outbreak described in the present study, the chronology of symptoms suggested that a 22-month-old infant was likely to be the primary case, which was notable as children under 4 years of age represented less than 1% of identified Irish SARS-CoV-2 infections at that time [12]. In a detailed analysis of 58 household clusters, Laws *et al.* observed secondary infection rates that were similar for both adults (30 %) and children (28 %), although children experienced symptoms less frequently than adults [13]. This contrasts with an analysis from Switzerland of familial clusters involving 39 paediatric SARS-CoV-2 infections, which showed that in only three cases (8 %) did a child develop symptoms before any other household contact [14] and several other reports which have also described an absence of transmission of SARS-CoV-2 from infants to their caregiver despite prolonged close contact [15–17]. However, it is unknown whether these findings represent a genuine difference in susceptibility to infection due to age-related factors, or if infants are being excluded from testing programmes due to their tendency to display milder symptoms [18, 19].

### SARS-CoV-2 cases co-infected with other respiratory viruses

As noted in this cluster, common respiratory viruses such as rhinovirus are not easily distinguished from SARS-CoV-2 infections on the basis of clinical symptoms alone. In fact, the presence of rhinorrhoea and sneezing caused by other viral pathogens could mask COVID-19 diagnosis, yet conceivably increase the propensity to spread concomitant SARS-CoV-2 infection by droplet and aerosol routes. Moreover, there is evidence emerging to suggest an upregulation of factors mediating SARS-CoV-2 entry across the human upper-airway epithelium, including the ACE2 receptor, by human rhinovirus A16 [20, 21]. Studies from early 2020 have estimated rates of SARS-CoV-2 co-infection with rhinovirus/enterovirus at 6.9 % [22]: as such, ongoing surveillance through the (northern hemisphere) winter season will be important to establish both the burden of these co-infections, and the impact they may have on SARS-CoV-2 attack rates.

### Role of WGS in SARS-CoV-2 outbreak investigations

Throughout the pandemic, WGS has played an important role in efforts to characterize the dynamics of transmission of SARS-CoV-2 within clusters and outbreaks. In the initial phase, genomic data generated in the context of a household outbreak provided important evidence for human-to-human transmission of the virus in China [8] and subsequently played a key role in the investigation of early cryptic transmission in the United States [23]. As the virus has spread and evolved, hundreds of thousands of SARS-CoV-2 genomes have been made available publicly on platforms such as GISAID [24], and such data has been key to informing public health policy development across many jurisdictions [25, 26]. It is noteworthy that a high proportion of mutations in the SARS-CoV-2 genomes correspond to C-to-U and A-to-G mutations, which are attributed to the effects of host-dependent RNA-editing proteins of the APOBEC and ADAR families, respectively [27]; the three mutations distinguishing the genome sequences derived from the family cluster from other sequences circulating in the region exemplify such effects.

In this cluster, WGS supplemented the epidemiological investigation with high-resolution data about the source and directionality of transmission of infection, as well as providing important background genomic data about locally circulating viruses. Firstly, it supported the entry, from a single point source, of infection into this family from genetically similar B.1 lineage viruses circulating in the south-east of the country; secondly, even in a setting whereby the history of events and chronology of symptoms pointed to case 1 as the primary case, WGS identified a genetically distinct viral sub-lineage from her sample that suggested she was infected later than other family members.

Although the identification of a spike substitution from the two children in this family outbreak cluster provided useful information about the chain of transmission between family members, the functional significance of this variant has yet to be established. This highlights the important role that WGS plays in tracking the genomic evolution of this virus and in informing public health responses, particularly in the context of widespread vaccine use. In recent months, a variety of important amino acid substitutions and deletions within the SARS-CoV-2 spike protein have been identified by genomic surveillance efforts in many countries with the potential to impact viral transmissibility, pathogenesis and/or facilitate immune-escape from neutralizing antibodies. For example, the B.1.1.7 ‘Variant of Concern’, which is defined by a set of 23 genomic variations including an S:N501Y substitution and deletions at positions 69–70, spread rapidly throughout the United Kingdom from late November 2020 [28] and received significant attention due to evidence supporting increased viral transmissibility and its association with the failure of S gene qPCR targets [28, 29]. Notable variants observed in Irish SARS-CoV-2 sequenced genomes between 25 June and 15 November 2020, are represented in Fig. 3 (a). These include D614G, associated with the B.1 lineage observed in this family cluster, which is now ubiquitous and appears to have a moderate effect on SARS-CoV-2 transmissibility [30]; A222V, present in the 20A.EU1 (B.1.177) cluster which has spread quickly across Europe from Spain and become dominant in Irish sequences [31]; and N439K, a variant for which there is evidence to suggest increased ACE2 receptor binding affinity and immune-escape from neutralising antibodies [32].

### Limitations of WGS

In this cluster, most family members were tested for SARS-CoV-2 by qRT-PCR on the same day, including case 1, and the majority had genetically indistinguishable viruses. Additionally, the moderate substitution rate in SARS-CoV-2 due to the replication with proofreading activity [33] limits the accumulation of mutations among samples. This meant that although a putative direction of transmission between the adult family members and case 1 could be hypothesized, the identity of the primary case could not be definitely proven. Although the possibility that more than one introduction of SARS-CoV-2 into the family cannot be out-ruled, the high nucleotide sequence similarity among the viral sequences recovered from the family members and the genetic distance to other sequences generated from contemporaneous community cases, indicated a single introduction into the family with spread between those in attendance at the gathering was the most parsimonious series of events. However, these points serve to highlight the potential limitations of WGS in this context; although it can add weight to hypotheses generated by outbreak investigations, directionality of transmission is not always possible to ascertain, and phylogenetic information must be taken in the context of metadata derived from a complete epidemiological evaluation.

## Conclusion

SARS-CoV-2 transmits readily within households and may be clinically indistinguishable from seasonal respiratory viruses. Genomic data from WGS provided high-resolution phylogenetic characterization of this household cluster, elucidating the chain of transmission between family members, and affording an insight into the source of infection from SARS-CoV-2 viral lineages in community circulation in Ireland. This underlines the important role that WGS can play as an aid to epidemiological investigations and efforts to better understand the transmission dynamics and genomic evolution of SARS-CoV-2.

## Supplementary Data

Supplementary material 1
